# GPER-1 acts as a tumor suppressor in ovarian cancer

**DOI:** 10.1186/1757-2215-6-51

**Published:** 2013-07-13

**Authors:** Tanja Ignatov, Saskia Modl, Maike Thulig, Christine Weißenborn, Oliver Treeck, Olaf Ortmann, AC Zenclussen, Serban Dan Costa, Thomas Kalinski, Atanas Ignatov

**Affiliations:** 1 Department of Obstetrics and Gynecology, Otto-von-Guericke University, G.-Hauptmann Str. 35, 39108, Magdeburg, Germany; 2 Department of Experimental Obstetrics and Gynecology, University Clinic, Magdeburg, Germany; 3 Department of Obstetrics and Gynecology, University Medical Center Regensburg, Regensburg, Germany; 4 Department of Pathology, University Clinic, Magdeburg, Germany

**Keywords:** GPR30, GPER-1, Ovarian cancer

## Abstract

**Background:**

It is known that the new membrane-bound estrogen receptor GPER-1 acts suppressive in breast cancer cells and its expression decreases during disease progression. This study was conducted to evaluate the GPER-1 expression in ovarian cancer and its correlation with progression. Its function was tested *in vitro* in ovarian cancer cells.

**Patients and methods:**

GPER-1 expression was analyzed by immunohistochemistry in 35 benign ovarian tumors, 35 tumors of low-malignant potential and in 124 ovarian cancers. GPER-1 expression was correlated to the prospectively evaluated disease-free survival of ovarian cancer patients. We also tested GPER-1 expression in ovarian cancer cells and the effect of GPER-1 stimulation on cell growth.

**Results:**

GPER-1 expression was significantly lower in ovarian cancer tissue than in benign and low-malignant ovarian tumors. GPER-1 expression was observed in 83.1% of malignant tumors and was higher in early stage cancers and tumors with high histological differentiation. GPER-1 expression was associated with favourable clinical outcome. The difference in 2-year disease-free survival by GPER-1 expression was significant, 28.6% for GPER-1 negative and 59.2% for GPER-1 positive cases (p = 0.002). GPER-1 expression was observed in SKOV-3 and OVCAR-3 ovarian cancer cell lines. G-1, a selective GPER-1 agonist, suppressed proliferation of the two cell types via inhibition of cell cycle progression in G2/M phase and stimulation of caspase-dependent apoptosis. The blockade in G2/M phase was associated with increased expression of cyclin B1 and Cdc2 and phosphorylation of histone 3.

**Conclusion:**

GPER-1 emerges as a new tumor suppressor with unsuspected therapeutic potential for ovarian cancer.

## Introduction

Ovarian cancer is a common neoplasm in Western countries and more than 70% of all diagnoses are in advanced stage [1,2]. The heterogeneity of ovarian cancer forces the researcher to find new strategies and targets to individualize the therapy of this common disease [3]. Recently the G-protein-coupled receptor 30 was claimed to be a new membrane-bound G-protein-coupled estrogen receptor-1 (GPER-1) involved in the rapid nongenomic effects of estrogen in normal and cancer tissue [4]. It belongs to the family of G-protein-coupled receptors (GPCRs) and acts independently of estrogen receptor (ER) α and ERβ [4]. GPER-1 stimulation is associated with increased cAMP production assuming a coupling of GPER-1 to Gαs [5]. Moreover, GPER-1 activation leads to activation and release of heparin-bound growth factor (HB-EGF), which in turn activates the epidermal growth factor receptor (EGFR) followed by phosphorylation of MAPK [6]. However, the physiological and pathophysiological function of GPER-1 remains incompletely discovered.

Despite the fact that GPER-1 can mediate the proliferative effects of estrogen in many estrogen-related cancers, we and others have demonstrated that GPER-1 can inhibit cell proliferation in different cell systems [7-11]. Moreover, we have recently found that GPER-1 down-regulation in breast cancer tissue is associated with poor clinical outcome [12].

The role of GPER-1 in ovarian carcinogenesis is only particularly and very controversial studied. Smith and co-workers have shown in 89 ovarian cancer patients that GPER-1 expression is associated with poor survival [13]. In contrast, Kolkova and co-workers have not found any correlation between GPER-1 expression and survival of 152 patients with ovarian cancer [14]. A third research group has found that GPER-1 expression predicts lower survival of 150 ovarian cancer patients only by co-expression with epidermal growth factor receptor (EGFR) [15]. *In vitro* studies provide also controversial data regarding GPER-1 effect on cell growth [16], adding confusion to the role of GPER-1 in ovarian cancer.

This study was undertaken to put more clearance in the role of GPER-1 in ovarian tumor biology. We investigated GPER-1 protein expression in benign and malignant ovarian tumors. GPER-1 expression was correlated to clinicopathological characteristics and clinical outcome of ovarian cancer patients, which was assessed prospectively. In addition, the effect of GPER-1 stimulation in ovarian cancer cells was assayed via its specific agonist G-1.

### Patients and methods

#### Patients and tissue samples

The data of 35 patients with benign ovarian tumors and 35 patients with ovarian tumors of low malignant potential (LMP), who had been admitted to the Department of Obstetrics and Gynecology, Otto-von-Guericke University, Magdeburg, Germany from 1999 to 2011, were selected by retrospective analysis. Additionally, tissue from 124 ovarian cancer patients was obtained at the operation in the Department of Obstetrics and Gynecology, University Clinic of Magdeburg, Germany in the period between 2005 and 2010. The study was approved by the Research and Ethical Committee of Otto-von-Guericke University, Magdeburg, Germany. The expression analysis of GPER-1 in ovarian cancer tissue was designed as a prospective monocentre cohort study. The primary outcome of the study was the correlation of GPER-1 expression and the 2-year disease-free survival (DFS) of ovarian cancer patients. Outcome was measured as DFS, according to the International Union Against Cancer (UICC) criteria [17]. Secondary outcome was the correlation of GPER-1 expression in ovarian cancer tissue and clinicopathological characteristics. The minimal follow-up period was 24 months. The inclusion criteria were diagnosis of ovarian cancer, no previous treatment with chemotherapy and no history of other carcinomas. Exclusion criteria included a previous history of adjuvant anti-hormonal or cytostatic therapy, incomplete adjuvant chemotherapy, no sufficient material for detection of GPER-1 expression and incomplete 24 months follow-up data. One hundred thirty seven eligible patients were enrolled in the study. Thirteen patients were secondary excluded because of lost of follow-up (7 patients), incomplete chemotherapy (3 patients) and detection of second cancer during the follow-up time (3 patients). All ovarian cancer patients underwent adjuvant platinum-based chemotherapy.

The median age at the time of primary diagnosis was 62 years (range 12–87 years) in the group of patients with benign ovarian tumors, 52 years (range 21–81 years) in the group of patients with LMP tumors and 64 years (range 20–86 years) in the group of patients with ovarian cancer.

#### Immunohistochemistry

GPER-1 expression was analyzed as previously described [18]. Briefly, sections of formalin-fixed and paraffin-embedded ovarian tumors, including benign ovarian cyst, tumors of low malignant potential (LMP) and malignant ovarian cancer specimens were investigated. The immunostaining was performed with affinity-purified rabbit antibody against GPER-1 (SP4677P; Acris antibodies, Herford, Germany) diluted 1:500. The slides were counterstained with hematoxylin and cover slipped after being embedded in mounting medium. The specificity of the antibody has been already proven [18]. Moreover, we assayed GPER-1 expression on 15 representative tissue samples of ovarian carcinoma with a second antibody against GPER-1 (sc-48524-R, Santa Cruz, Heidelberg, Germany diluted 1:500). The obtained expression pattern with the two antibodies was very similar and confirms again their specificity (data not shown).

**Figure 1:**
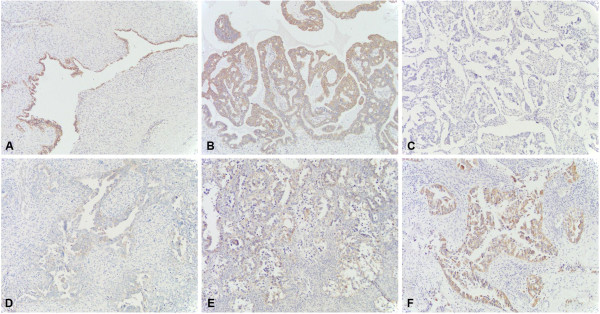
**Paraffin-embedded ovarian tumor tissue immunostained with GPER-1 antibody.** GPER-1 immunostaining of ovarian cancer tissue: **(A)** strong positive expression in benign ovarian tumor; **(B)** strong positive expression in tumor of low malignant potential; **(C)** negative, **(D)** weak, **(E)** moderate and **(F)** strong positive staining of GPER-1 in ovarian cancer tissue. Original magnification: x 100.

GPER-1 expression was classified as already described [18,19], according to the following grading system: staining extensity was categorized as 0 (no positive cells), 1 (<10% positive cells), 2 (10-50% positive cells), or 3 (>50% positive cells), and staining intensity was categorized as 0 (negative), 1 (weak), 2 (moderate), or 3 (strong). The individual categories were multiplied to give a total immunohistochemical score (IHC). IHC score ranged between 0 and 9. GPER-1-positive expression was defined for tumors that showed an IHC ≥1. Representative example of GPER-1 expression is shown in Figure 1.

#### Cell culture and treatment

Ovarian cancer cell lines SKOV-3 and OVCAR-3 were obtained from Cell Lines Services (Germany) and routinely cultured in DMEM/F12 (PAN Biotech) supplemented with 10% FBS, 100U/ml penicillin and 100 μg/μl streptomycin at 37°C in a humidified 5% CO_2_ atmosphere. The cells were treated as indicated in the figure legends.

#### Western blotting

Western blotting procedures were performed as previously described [18]. Briefly, the blots were incubated overnight at 4°C with antibodies against GPER-1 (sc-48524-R, Santa Cruz, Heidelberg, Germany, diluted 1:500), caspase-3 (ab13847, Abcam, Cambridge, UK, diluted 1:1000), cyclin B1(#4138, Cell signalling, Frankfurt am Main, Germany, diluted 1:1000), Cdc2 (#9112, Cell signalling, Frankfurt am Main, Germany, diluted 1:2000) phosphoH3(Ser10) (#9701, Cell signalling, Frankfurt am Main, Germany, diluted 1:2000) and for 2 h at room temperature with antibody against β-actin (A5441, Sigma Aldrich, Hamburg, Germany diluted 1:10000). Peroxidase-conjugated anti-rabbit and anti-mouse antibodies (Thermo Scientific, Waltham, MA, USA), diluted 1:2000 and 1:5000, were used as secondary antibodies (2 h at room temperature), respectively.

#### Cell cycle analysis and apoptosis assay

Cell lines were treated with 1 μM G-1 or vehicle control for 48-72 h. Cell cycle distribution was analysed by propidium iodide (PI) staining using flow cytometry. Apoptosis was determined by using the FITC Annexin V Apoptosis Detection Kit (BD, Heidelberg, Germany).

#### MTT-viability assay

MTT-viability assay was performed as already described [20]. Briefly, 2000 cells per well were seeded and cultured in 96-well plate in growth medium. After 24 h the cells were stimulated with indicated concentrations of G-1, G-15 or DMSO as control for 5 days. This was followed by a 3 h treatment with MTT (3-[4,5-dimethylthiazol-2-yl]-2,5-diphenyltetrazolium bromide) in the dark at 37°C. After removing the supernatants and incubation in 150 μl lysis buffer (isopropanol containing 4 mM HCl and 0,1% NP-40 ) cell viability was determined by measuring the absorbance at 570 nm (Synergy HT, BioTek, Germany).

### Statistical analysis

The statistical calculations were performed using SPSS Version 20.0 (SPSS, Chicago, IL, USA). An association between GPER-1 expression and the tumor variables was evaluated using the x^2^ test or Fisher’s exact test. DFS analysis took into account those who died of ovarian cancer-specific death or had a recurrence of disease as a primary event. Patients who died of other causes were censored. Survival was calculated using the Kaplan-Meier method. The equality of survival curves was tested by the log rank test. Univariate Cox proportional hazard regression analysis was used to identify significant prognostic factors. The prognostic significance was confirmed by multivariate analysis. The statistical analyses were two sided and p-values of <0.05 were considered statistically significant. The comparison of GPER-1expression between benign tumors, LMP tumors and ovarian cancers was analyzed using nonparametric paired test, and was performed with the Wilcoxon signed rank sum test.

## Results

### GPER-1 protein expression in ovarian tumors and correlation with clinical and pathologic features of ovarian cancer

**Figure 2:**
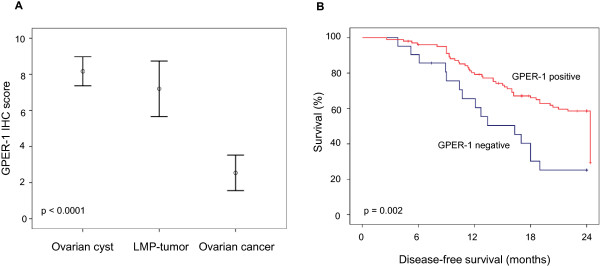
**GPER-1 protein expression and clinical outcome. A)** Protein expression of GPER-1 in ovarian tumors. **B)** Disease-free survival of ovarian cancer patients according to GPER-1 expression. The log rank test was used to calculate the p*-*value.

GPER-1 expression was assessed by immunohistochemistry in 35 benign ovarian tumors, 35 ovarian tumors of low malignant potential (LMP) and 124 ovarian cancers. GPER-1 expression was observed in all (100%) benign ovarian tumors, in 34 of 35 (97.1%) tumors of LMP and in 103 of 124 (83.1%) of ovarian cancers. GPER-1 expression was significantly lower in ovarian cancer compared to the benign and tumors of LMP (p < 0.0001; Figure 2A). The median IHC-score was 6 for benign and tumors of LMP, whereas ovarian cancer tissue demonstrated a median IHC-score of 2.

Next, we compared GPER-1 protein expression with the clinicopathological features of ovarian cancer. GPER-1 expression was significantly associated with the clinical stage of disease (p = 0.032; Table 1). Loss of GPER-1 expression was more frequently observed in advanced ovarian cancer. Moreover, a reduction of GPER-1 expression of approximately 20% was observed with decrease of histological differentiation (Table 1). However, this correlation did not reach a significant level. There was no correlation between GPER-1 immunostaining and other tumor characteristics (Table 1). All this data suggested a loss of expression of GPER-1 during ovarian tumorigenesis.

**Table 1:** Characteristics of study cohort

**Characteristic**	**N of patients**	**GPER-1**		**p-value**
		**Negative**	**Positive**	
		**N (%)**	**N (%)**	
Total	124	21 (16.9)	103 (83.1)	
Age, years (range)	124	65 (30–84)	61 (20–85)	0.136
Menopausal status				
premenopausal	16	1 (6.2)	15 (93.8)	
postmenopausal	108	20 (18.5)	88 (81.5)	0.304
FIGO Stage				
1	25	0 (0)	25 (100)	
2	21	6 (28.6)	15 (71.4)	
3	76	14 (18.4)	62 (81.6)	
4	2	1 (50.0)	1 (50)	0.032
Lymph node status				
negative	31	5 (16.1)	26 (83.9)	
positive	18	3 (16.7)	15 (83.3)	1.000
Histological grading				
1	18	0 (0)	18 (100)	
2	38	7 (18.4)	31 (81.6)	
3	68	14 (20.6)	54 (79.4)	0.112
Histology				
serous	81	13 (16.0)	68 (84.0)	
mucinous	16	3 (18.8)	13 (81.2)	
endometrioid	13	2 (15.4)	11 (84.6)	
clear cell	8	0 (0)	8 (100)	
other	6	3 (50)	3 (50)	0.171
Recurrence (in 24 months)				
no	57	4 (7.0)	53 (93.0)	
yes	67	17 (25.4)	50 (74.6)	0.008

### GPER-1 expression and clinical outcome of ovarian cancer patients

During the prospective follow-up time of 24 months we observed 67 recurrences of cancer. There was a significant difference in DFS between GPER-1-positive and GPER-1-negative patients (Table 1). GPER-1 positivity was associated with reduced rate of disease recurrence (p = 0.008).

Univariate analysis revealed that GPER-1 expression was a favorable prognostic factor for DFS (HR, 0.404; 95% CI, 0.233-0.732; *p* = 0.003) (Table 2). The 2-year DFS was 28.6% for GPER-1-negative patients and 59.2% for GPER-1-positive patients (p = 0.002; Figure 2B). After adjustment for patient age, menopausal status, tumor stage, lymph node status, histological grading, multivariate analysis rendered GPER-1 as an independent, favorable prognostic factor in regard to DFS (HR, 0.569; 95% CI, 0.317-0.991; p = 0.046) (Table 2). Tumor stage was evaluated as an unfavorable independent prognostic factor (HR, 2.764; 95% CI, 1.590-3.751; p < 0.0001).

**Table 2:** Uni- and multivariate analysis in regard to DFS

**Variable**	**HR**	**95% CI**	**P-value**
**Univariate analysis**			
GPER-1 expression	0.404	0.233-0.732	0.003
Age	1.010	0.990-1.031	0.322
Menopausal status	1.676	0.715-3.930	0.235
Tumor stage	3.023	1.774-5.150	<0.0001
Lymph node status	1.950	0.731-5.197	0.182
Histological grading	2.132	1.363-3.335	0.001
**Multivariate analysis**			
GPER-1 expression	0.549	0.317-0.991	0.046
Tumor stage	2.764	1.590-3.751	<0.0001
Histological grading	1.576	0.982-2.531	0.060

### Effect of GPER-1 stimulation on ovarian cancer cell proliferation

**Figure 3:**
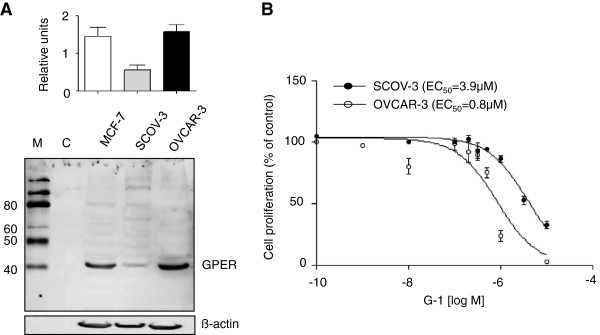
**GPER-1 expression and G-1 effect on ovarian cancer cells. A)** Representative example of GPER-1 protein expression in MCF-7 breast cancer cells and in SCOV-3 and OVCAR-3 ovarian cancer cells. ß-actin was used as a loading control. **B)** SCOV-3 and OVCAR-3 cells were treated with indicated concentration of G-1 and the cell number was counted using MTT viability assay. Comparison of the dose–response curves yielded IC_50_ values of 3.9 μM and 0.8 μM for SCOV-3 and OVCAR-3, respectively. Each experiment was repeated at least three times. The results are expressed as means ± SD. M, marker; C, negative control (H2O).

Next we wanted to test the effects of GPER-1 *in vitro*. First of all, we analyzed the protein expression of GPER-1 in SKOV-3 and OVCAR-3 ovarian cancer cells by western blot (Figure 3A). MCF-7 breast cancer cells were used as positive control. GPER-1 expression was observed in the two ovarian cell lines. However, OVCAR-3 cells demonstrated a 2-fold higher GPER-1 expression than SKOV-3 cells. Then we investigated whether GPER-1 influences the growth of ovarian cancer cells. Both ovarian cell lines were incubated with increasing concentrations of GPER-1 specific agonist G-1. A concentration-dependent inhibition of cell proliferation was observed (Figure 3B). The estimated IC_50_ value was 3.9 μM and 0.8 μM for SKOV-3 and OVCAR-3 cells, respectively.

**Figure 4:**
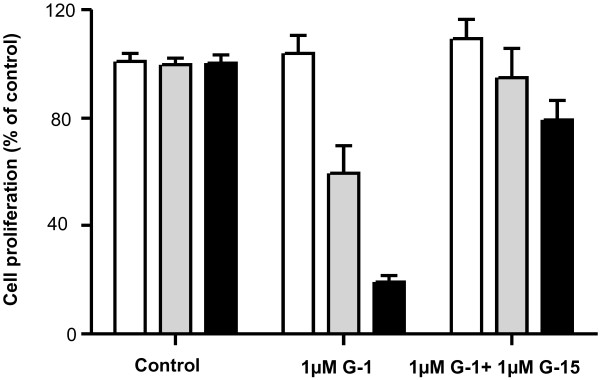
**GPER-1 specific antagonist G-15 abolished the G-1-induced inhibitory effect in ovarian cancer cells.** GPER-1-positive SCOV-3 and OVCAR-3 ovarian cancer cells and GPER-1-negative HEK-293 cells were incubated for 5 days with 1 μM G-1 with or without pre-treatment with 1 μM G-15 for 24 h and the cell number was counted using MTT viability assay. Each experiment was repeated at least three times. The results are expressed as means ± SD.

Furthermore, we wanted to exclude a possible cytotoxic effect of high G-1 concentrations. For this purpose we incubated SKOV-3, OVCAR-3 and GPER-1-negative HEK-293 cells with 1 μM G-1 for 5 days. As demonstrated in Figure 4, G-1 did not change the proliferation of HEK-293 in comparison to control-treated cells. It suggested that G-1 inhibitory effect in SKOV-3 and OVCAR-3 ovarian cancer cells due to specific activation of GPER-1 and not to cytotoxic properties. To explore the involvement of GPER-1 activation in G-1-induced cell growth inhibition we extended our experiments using G-15 a recently discovered GPER-1 receptor antagonist [21]. Pre-incubation of SKOV-3 and OVCAR-3 cells with 1 μM G-15 was able to block the inhibitory effect of G-1 (Figure 4). As expected in GPER-1-negative HEK-293 cells G-15 pre-incubation was not associated with any changes in cell proliferation (Figure 4). All these data suggested an involvement of GPER-1 in G-1-induced proliferation effects.

### GPER-1 activation by G-1 induced a cell-cycle arrest and cell apoptosis in SKOV-3 and OVCAR-3 cells

**Figure 5:**
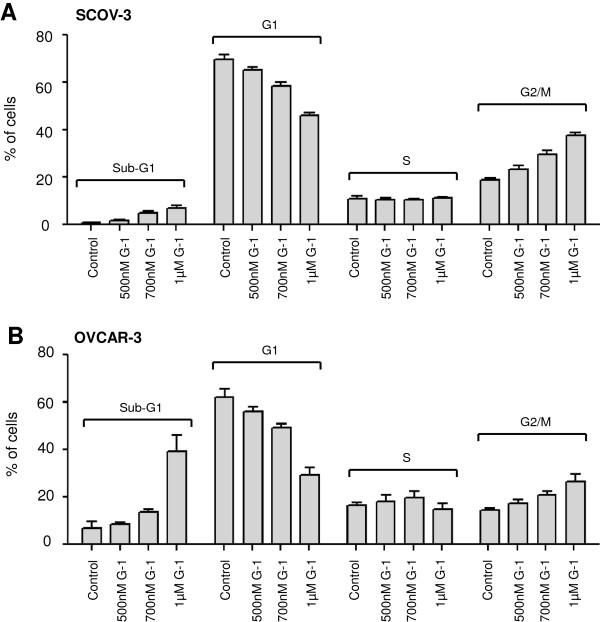
**Effects of G-1 on cell cycle progression in ovarian cancer cells.** Cell cycle distribution of **(A)** SCOV-3 and **(B)** OVCAR-3 cells treated or not with indicated concentrations of G-1 for 48 h was determined by flow cytometry analysis. Each experiment was repeated at least three times. The results are expressed as means ± SD.

We next tested by flow cytometry analysis whether GPER-1 activation by G-1 results in abrogation of cell cycle progression. Stimulation of ovarian cancer cells with G-1 led to concentration-dependent accumulation of SKOV-3 (Figure 5A) and OVCAR-3 (Figure 5B) cells in apoptotic sub-G1 phase and decreased the cell number in G1 phase. Higher concentrations of G-1 were associated with additional block in G2/M phase.

**Figure 6:**
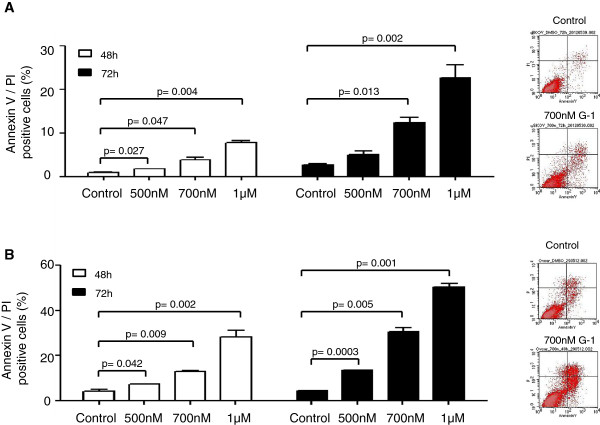
**G-1 induces apoptosis in ovarian cancer cells. A)** SCOV-3 and **B)** OVCAR-3 cells were treated with control or with indicated concentrations of G-1 for 48 h, afterwards cells were stained with annexin V/propidium iodide and analyzed by FACS flow cytometry. Each experiment was repeated at least three times. The results are shown as means ± SD. Representative examples of distribution of SCOV-3 and OVCAR-3 cells treated with control (upper panel) or 700 nM G-1 (lower panel) is shown.

To confirm the effect of GPER-1 on cell apoptosis AnnexinV assay was applied. A concentration- and time-dependent increase of cell apoptosis was observed in the two cell lines after stimulation with G-1 (Figure 6). In the SKOV-3 cell line we observed a concentration-dependent increase of cell apoptosis by 1%, 3% and 7% after 48 h stimulation with 500 nM, 700 nM and 1 μM G-1, respectively (Figure 6A). After 72 h incubation duplication of G-1-induced cell apoptosis was observed. In OVCAR-3 cells G-1 treatment caused even higher rates of cell apoptosis. After treatment with G-1 for 48 h in aforementioned concentrations cell apoptosis reached a level of 7% 13%, and 28%, respectively (Figure 6B). After 72 h stimulation the rate of cell apoptosis increased to 13%, 30%, and 49% after incubation with 500 nM, 700 nM and 1 μM G-1 respectively (Figure 6B).

**Figure 7:**
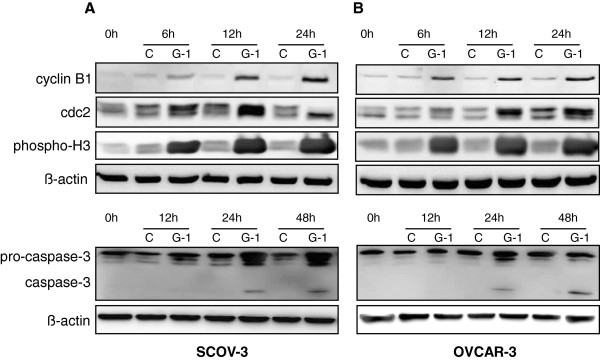
**GPER-1 specific agonist G-1 induced expression of cyclin B1 and Cdc2 regulatory proteins, phosphorylation of histone 3 and cleavage of pro-caspase-3.** SCOV-3 **(A)** and OVCAR-3 **(B)** ovarian cancer cells were incubated with medium (control) or 1 μM G-1 for indicated times and the protein expression of cyclin B1, Cdc2 and caspase-3 as well as the phosphorylation of histone 3 were investigated by western blot analysis. ß-actin was used as a loading control. Each experiment was repeated three times.

These data suggested that GPER-1 might inhibit ovarian cancer cell proliferation via simultaneous cell cycle arrest and cell apoptosis. To investigate the G-1-induced cell cycle arrest and apoptosis in more details we examined the expression of different regulatory proteins involved in cell cycle progression and apoptosis. After stimulation of SCOV-3 (Figure 7A) and OVCAR-3 (Figure 7B) cells with 1 μM G-1 for indicated time, the expression of cyclin B1 and Cdc2, the phosphorylation of histone H3 and the cleavage of caspase-3 was measured by immunoblotting. In the two cell lines tested in this study, we found that G-1-induced cell cycle arrest and apoptosis was associated with up-regulation of cyclin B1 and Cdc2 protein expression. The phosphorylation of histone H3 was also observed (Figure 7). The levels of cyclin B1 and Cdc2 protein expression started to increase at 6 h after G-1 treatment, reached a peak between 12 and 24 h followed by rapid decline (data not shown). The phosphorylation of histone H3, indicative for cells in mitotic phase, started to increase at 6 h after stimulation of the cells with G-1 and peaked at 12 h (Figure 7).

The caspase-3 cleavage product as result of its activation was observed in both cell lines after incubation with G-1. The activation of caspase-3 started at 24 h after GPER-1 activation and peaked around 48 h in the two cell lines (Figure 7).

## Discussion

This is the first report demonstrating the favourable role of GPER-1 in ovarian cancer patients in regard to disease-free survival (DFS). GPER-1 expression and clinical outcome were investigated in a very homogenous cohort of platinum-treated ovarian cancer patients. GPER-1 expression was a favourable factor regarding 2-year DFS in ovarian cancer patients. Moreover, GPER-1 expression decreased from benign to malignant ovarian tumours. *In vitro*, GPER-1 stimulation was associated with a significant increase of cellular apoptosis and cell cycle arrest in ovarian cancer cells.

To date there are only few reports about GPER-1 and clinical outcome of ovarian cancer patients providing very confusing and controversial results. Smith and co-workers have recently found that GPER-1 expression predicts poor survival of ovarian cancer patients [13], which is in the opposite to our observation. In this report the median overall survival was significantly shorter for patients expressing high levels of GPER-1. It is remarkable, that this correlation was observed only in adverse tumors but not for low grade tumors. Unfortunately, the impact of GPER-1 on patient outcome has not been confirmed via multivariate analysis. In the aforementioned patient collective GPER-1 expression significantly correlated with tumor stage and tumor grading, two known prognostic factors in ovarian cancer patients, which could explain the impact of GPER-1 on survival. In our cohort of patients GPER-1 was independent favourable prognostic factor even after adjustment of other prognostic factors. Another plausible explanation of the controversial results is a difference in population studied. Interestingly, another research group has not found any correlation between GPER-1 expression on mRNA and protein level with clinical outcome of ovarian cancer patients [14]. It is to note that in this cohort of 150 patients GPER-1 was expressed in only one third, whereas in our cohort more than 80% of the invasive ovarian cancers expressed GPER-1. The difference might be explained by the specificity of the antibodies used, the evaluation criteria of GPER-1 expression or by the fact that Kolkova et al. correlated GPER-1 expression only to overall survival but not to DFS [14]. Similar results were observed in analysis of 152 patients by Fjiwara et al. [15]. In these series GPER-1 expression has not shown any correlation with overall survival of ovarian cancer patients. However, the authors stated that EGFR expression in combination with GPER-1 predicts lower survival in patients with ovarian cancer. This is in agreement with our recent results demonstrating that GPER-1 cross-talks with EGFR in breast cancer cell lines [22]. The cross-talk between GPER-1 and EGFR is associated with activation of completely different signalling pathways [5,6,23] and could be responsible for the observed difference in survival rates. In agreement with the results obtained by us in the present study we have recently found that GPER-1 is associated with better patient survival in breast cancer patients [18] and have been recently confirmed for inflammatory breast cancer [24].

In accordance to expressional analyses, *in vitro* studies revealed controversial results regarding cellular functions of GPER-1. Since establishment of GPER-1 as an estrogen receptor many reports demonstrated its stimulatory effect in breast, ovarian and endometrial cells [23,25,26]. Nevertheless, numerous recent investigations clearly demonstrated an inhibitory effect of GPER-1 in various cell systems [7-11,16,27] and are in agreement with our findings. G-1 inhibitory effect in these cells systems has been observed only in high concentrations similar to our results in ovarian cancer cells. The absence of G-1-induced inhibition in GPER-1-negative HEK293 cells exclude a potential cytotoxic effect to the cells.

GPER-1 stimulation inhibited ovarian cell proliferation by inducing cell apoptosis and cell cycle arrest. We observed an accumulation of cyclin B1 and Cdc2 regulatory proteins in G-1-treated ovarian cancer cells. This is contradicting the results observed in breast and prostate cancer cells where cyclin B1 and Cdc2 expression were inhibited by G-1 stimulation [9,10]. Possible explanation for this discrepancy could be the use of different cell types or the fact that breast cancer cells and prostate cancer cell were blocked in G1 [9] and G2 phase [10], respectively. The suppression of Cyclin B1 and Cdc2 is associated with G2 arrest, whereas the up-regulation of the two proteins induced a cell accumulation in the M phase [28]. Thus the G-1-induced activation of Cyclin B1 and Cdc2 in ovarian cancer cells is associated with progression in the G2 phase to M phase. It was confirmed by the observation that phosphorylation of histone H3, an important step occurring during G2 to M transition [29], was significantly induced by G-1 activation. The perturbation of mitotic progression by GPER-1 stimulation with consequent increased mitotic duration could trigger caspase-3 cleavage and cell apoptosis. This phenomenon has been already described for taxol and nocodazole, two microtubule inhibitors with anticancer activity [28,30]. The inhibitory effect of GPER-1 in ovarian cancer cells has been recently well documented and is in accordance to our results. Henic and co-workers have recently found that GPER-1 stimulation attenuates the invasive properties of ovarian cancer cells [31].

Our results and the review of literature make us to hypothesize that GPER is a potential tumor suppressor. Tumor suppressors are known to be inactivated during cancer progression [32] which was also observed for GPER-1. The decreased expression of GPER-1 benign and malignant ovarian tumors corroborates the presumption that GPER-1 might be a tumor suppressor. This phenomenon was observed by us during the breast cancer tumorigenesis and was confirmed by two other groups [33,34]. We have found that GPER-1 is inactivated via promoter methylation in breast cancer tissue and is re-expressed after treatment with de-methylating agent 5-Aza (own not published data).

The most important finding of our study is the fact that GPER-1 with its high expression of 80% in ovarian cancer tissue could be an appropriate target for individualized therapy of ovarian cancer. Our study provides evidence that GPER-1 may be a tumor suppressor gene. First, GPER-1 specific agonist inhibited ovarian cancer cell proliferation by inducing cell apoptosis and partially cell cycle arrest. Second, GPER-1 expression was found to be down-regulated during ovarian cancer tumorigenesis.

## Competing interests

The authors declare that they have no competing interests.

## Authors’ contributions

TI carried out part of the evaluation of the IHC analysis, western blot analysis and statistical analysis, participated in the design of the study and analysis of the clinical data and drafted the manuscript; SM carried out the *in vitro* assays, MT participated in IHC analysis, CW carried out part of *in vitro* assays; OT, OO, AZ, SDC participated in study design and analysis of clinical data; TK and AI carried out part of IHC analysis, participated in the design of the study and statistical analysis of the clinical data, contributed methodological knowhow and drafted the manuscript. All authors read and approved the final version of the manuscript. All authors read and approved the final manuscript.
